# Complete genome sequence of *Mycobacterium smegmatis* phage Rummer, a subcluster A3 actinophage

**DOI:** 10.1128/mra.01268-23

**Published:** 2024-03-11

**Authors:** Dondra S. Bailey, Dominique R. Dotson, Charlotte Berkes, Oluwanifemi Agbede, Marcelaine Augustin, Imani Blackman-Murray, Talaeya Chambers, Nicolas Felber, Dy'Mon Fleming, Loretta Frazier, Natalie Gray, Ayanna Harrison, Genesis Hernandez, Nina Iwuchukwu, Chika Iwuji, Taysha Jackson, Angelic Jefferson, Daya Jordan, Miracle Jordan, Brian Nicolas, Monae Person, Ga'Nayah Richardson, Ashley Roman, Christian Stevens, My'Sean Suggs, Nahshon Thompson, Summer Timmons-Smith, Shiaishea Wilfong, Micaela Wilson-Wheatley

**Affiliations:** 1 Department of Natural Sciences, Coppin State University, Baltimore, Maryland, USA; 2 Department of Biology, Merrimack College, North Andover, Massachusetts, USA; Portland State University, Portland, Oregon, USA

**Keywords:** bacteriophage, actinophage

## Abstract

Bacteriophage Rummer is a siphovirus morphology actinophage isolated from *Mycobacterium smegmatis*. Rummer has a 50,908 base pair genome encoding 89 predicted protein-coding genes and three tRNAs. Based on gene content similarity to sequenced actinobacteriophages, Rummer is assigned to phage subcluster A3.

## ANNOUNCEMENT

The characterization of novel *Mycobacterium* bacteriophages may advance the development of therapeutics for *Mycobacterium leprae* and *Mycobacterium tuberculosis* infections (1). Here, we present the characterization and genome sequence of an actinophage, Rummer, infecting *Mycobacterium smegmatis*.

**Fig 1:**
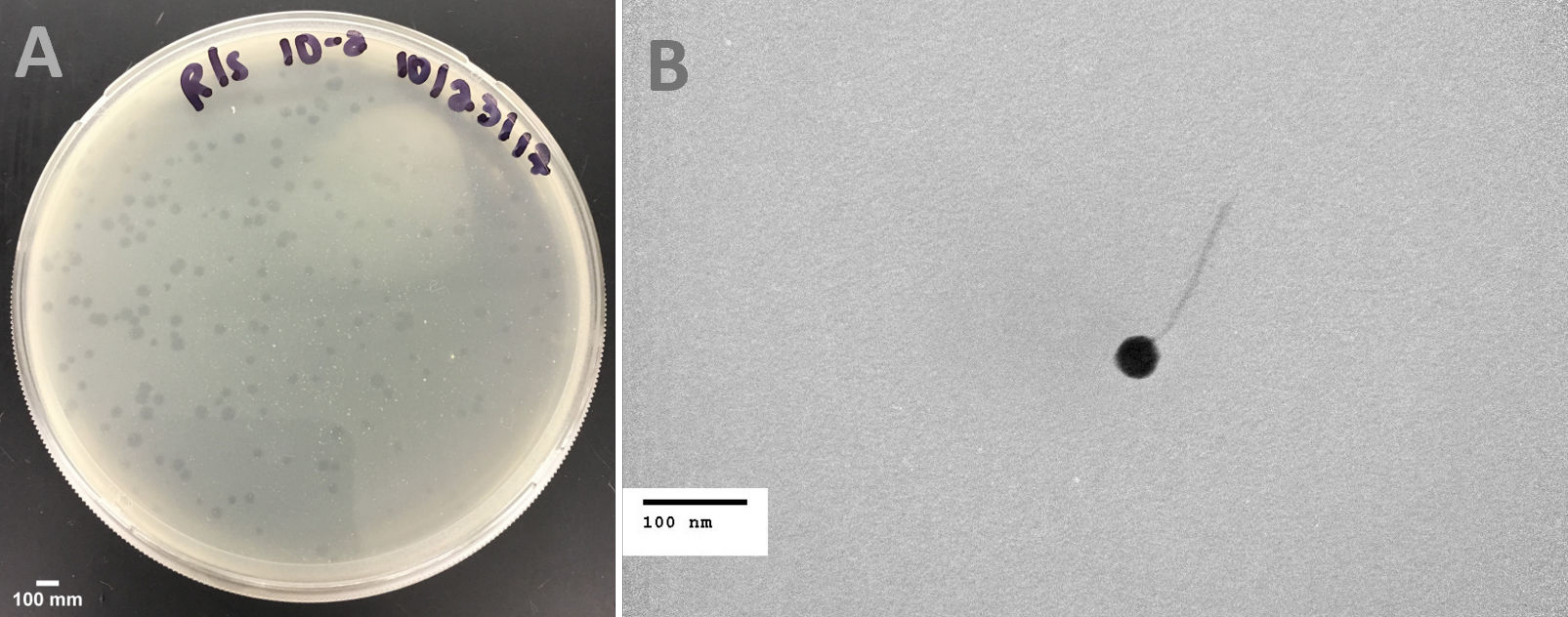
Characterization of Rummer. (**A**) Plaque morphology. Plaques produced on a lawn of *M. smegmatis* PYCa medium on a petri dish (90 mm diameter). Plaques are with a clear center surrounded by a turbid edge. (**B**) Negative-stain (1% uranyl acetate) transmission electron micrograph of Rummer. Phage particles were analyzed using JEOL JEM-1400 Transmission Electron Microscope at an accelerating voltage of 80 kV. Rummer has a siphoviral morphology with a capsid diameter of 50 nm and a tail length of 160 nm (*n* = 1).

Rummer was isolated in September 2017 from soil collected on the campus of Merrimack College in North Andover, MA (global positioning system coordinates 42.6686 N, 71.1216 W) using standard procedures (2). Briefly, the soil was incubated in liquid 7H9 media for 2 hours at 37°C, with shaking, and filtered through a 0.2 µm filter. The filtrate was added to a liquid culture containing late log-phase *Mycobacterium smegmatis* mc^2^155 and incubated for 24 hours at 37°C, with shaking. The liquid fraction of the overnight culture was filtered (0.2 µm pore size) and the filtrate was spotted on a lawn of *M. smegmatis* in top agar. Rummer was plaque-purified from the filtrate via three rounds of plating, with plates incubated at 37°C for 24 hours. Rummer plaques were turbid in appearance and approximately 2 mm in diameter (Fig. 1A). Analysis of phage particles by negative-stain transmission electron microscopy revealed that Rummer has a siphovirus morphology with a capsid diameter of 50 nm and a tail length of approximately 160 nm (*n* = 1, Fig. 1B).

Genomic DNA extraction was performed from filtered high-titer lysates using the Promega Wizard DNA kit. A sequencing library was prepared using the NEBNext Ultra II FS kit and the genome was sequenced using an Illumina MiSeq (v3 reagents), yielding 722,374 150-base single-end reads and 2,012-fold coverage. Raw reads were assembled and checked for completeness using Newbler v2.9 and Consed v29 (3, 4). The resulting genome has a 10-base 3´ single-stranded overhang (CGGGTGGTAA), a G + C content of 64.1%, and a length of 50,908 base pair. An automated annotation was generated using DNA Master (cobamide2.bio.pitt.edu; v5.23.6, build 2705, 24 October 2021), Glimmer (v3.02b) (5), and GeneMark (6), and start sites manually refined utilizing Starterator (http://phages.wustl.edu/starterator/) and Phamerator (7). Functions for putative genes were assigned using the PECAAN (discover.kbrinsgd.org) platform and based on Phagesdb Function Frequency, Phagesdb BLAST, and NCBI BLAST (Blastp from NCBI blast version 2.13.0+) (8) and HHPRED (against the PDB mmCIF70, Pfam-A, and NCBI Conserved Domain databases) (9). Three tRNAs were identified using ARAGORN (v1.2.41) (10) and tRNAscan-SE (v2.0) (11). Default parameters were used for all programs.

Eighty-nine putative genes were identified as protein-coding genes, of which 43 could be assigned putative functions. Based on gene content similarity of >35% to other phages in the Actinobacteriophage Database, PhagesDB (12, 13), Rummer was assigned to cluster A, subcluster A3, which all share the following characteristics: 3´ single-stranded genome ends, structural proteins encoded located on one-half of the genome and transcribed in the forward direction while DNA metabolism functions are encoded on the other half and transcribed in the reverse direction. A noteworthy feature of Rummer is that it is in a subset of phages A3 subcluster phages (13%) that encode three tRNAs. As with a majority of cluster A phages, Rummer encodes putative immunity repressor and integrase functions, suggesting it is a temperate phage.

## Data Availability

Rummer is available at GenBank with accession no. OR725494.1 and sequence read archive (SRA) no. SRX22853657.
